# Immediate Effectiveness of Chest Proprioceptive Neuromuscular Facilitation (PNF) Technique on Hemodynamic Status in COVID-19 Patients: An Original Research

**DOI:** 10.7759/cureus.29477

**Published:** 2022-09-22

**Authors:** Pallavi R Bhakaney, Rashmi R Walke, Chaitanya A Kulkarni, Vishnu Vardhan

**Affiliations:** 1 Department of Cardiorespiratory Physiotherapy, Datta Meghe Institute of Medical Sciences, Wardha, IND; 2 Department of Community Physiotherapy, Datta Meghe Institute of Medical Sciences, Wardha, IND; 3 Department of Cardiorespiratory Physiotherapy, Ravi Nair Physiotherapy College, Datta Meghe Institute of Medical Sciences, Wardha, IND

**Keywords:** rehabilitation, intercostal stretch, covid-19, chest pnf, anterior basal stretch lift

## Abstract

Introduction

COVID-19 is an infectious illness that first appeared in Wuhan, China in December 2019, and subsequently spread over the entire world. Those who were affected suffered from fever, cough, weakness, and breathlessness, along with the probability of developing pneumonia which sometimes leads to respiratory failure. The older population with co-morbidities was at higher risk.

Methods

In this experimental study, 59 subjects with COVID-19 were included according to pre-decided inclusion and exclusion criteria. Pre-vitals before the treatment were noted such as heart rate, respiratory rate, and oxygen saturation (SPO_2_), and then the chest proprioceptive neuromuscular facilitation (PNF) technique was given as a treatment regime, and its immediate effect was calculated. Post-vitals were noted immediately after the treatment session. The statistical analysis was made using the student's paired t-test.

Results

We found a mean difference of 9.45±5.27 in heart rate before and after the intervention provided which is suggestive of a statistically significant increase within normal limits. Also, the mean value of respiratory rate immediately after the intervention was shown as 22.91 and pre-intervention was 19.18 with a mean difference of 2.93±3.95, showing a statistically significant increase in respiratory rate. Additionally, the mean value of SPO2 immediately after the intervention was shown as 92.76 and pre-intervention was 94.64 and had a mean difference of 1.88±1.67 suggestive of high improvement in oxygen saturation immediately after the intervention.

Conclusion

The present study concludes from the available statistical analysis that chest PNF techniques such as intercostal stretch and anterior basal stretch lift have an immediate impact on improvement in oxygen saturation in COVID-19 patients and can be incorporated as an inpatient treatment regime in the rehabilitation protocol.

## Introduction

Coronavirus disease (COVID-19) is caused by a variant of several acute respiratory syndromes (SARS) [1]. First reported and spread to the nations globally, SARS-CoV-2 belongs to a β-coronavirus family and has a virtually similar genomic sequence to a bat coronavirus recognized as the bat is a natural domicile which is then transferred to humans [2]. It predominantly affects the respiratory tract and utilizes ACE2 (angiotensin-converting enzyme). Fever, dyspnea, coughing, lethargy, and occasionally gastrointestinal issues are among the disease's clinical signs. The most adverse symptoms are seen in the adult population who presented with a history of cardiovascular disease, endocrine disease, and metabolic diseases [3]. These may be associated with pneumonia which can progress and lead to acute respiratory distress syndrome (ARDS) [4-6]. Complimentary and integrated health care approaches have shown improvement in resilience and immune responses among people [7]. To effectively prevent additional disease progression, lessen the risk of severe post-recovery disability, and to increase respiratory muscle function in order to improve breathing during exercise, early evaluation and rehabilitation interventions are required for COVID-19 patients [8]. Interventions include breathing exercises which are aimed at improving the respiratory muscles' capacity and coordination. The capacity of the thorax, which is controlled by the flexibility of the skeletal muscles, the pliability of the nearby soft tissues, and the strength of the respiratory muscles, has an influence on the expansion and contraction of the lungs. The utmost effort made by the muscles involved in chest expansion during breathing determines the strength of the respiratory muscles. Through resistance training with proprioceptive neuromuscular facilitation (PNF), muscle strength can be increased. It gives the respiratory muscles proprioceptive feedback, which triggers reflex respiratory movement responses and enhances the rate and depth of breathing. Repeated compressions are performed to promote an improvement in inspiratory volume, while a stretch response is used to facilitate the onset of inhalation [9]. This effect could lead to a beneficial effect in COVID-19 patients with reduced functional capacity and chest wall mobility. Thus, the study was aimed at determining the immediate effectiveness of the chest PNF technique on the hemodynamic status of COVID-19 patients.

## Materials and methods

The study was done after approval from the Institution Ethical Committee of Datta Meghe Institute of Medical Sciences with the approval number DMIMS(DU)/IEC/2021/352. A total of 59 COVID-19-positive patients were included in the study. The patients were hospitalized in the COVID-19 ICU of Shailinitai Meghe Superspeciality Hospital. It was an interventional study type. Patients who fulfilled the inclusion criteria - positive COVID-19 patients who were admitted to COVID ICU and with high flow oxygen support, with no co-morbidities, aged 21 years and above, and those affected mildly and moderately according to the CT severity score - were incorporated into the study. Patients' informed consent was also obtained. All the patients tested positive for reverse transcription polymerase chain reaction (RT-PCR) and were getting medical treatment for COVID.

Before commencing the treatment, baseline heart rate, respiratory rate using ECG leads, and oxygen saturation were taken. The vitals of the patients were monitored throughout the training program. The exercises given were tailor-made and performed according to the patients’ comfort and hemodynamic stability.

The patients were given a combination of two chest PNF techniques that is intercostal stretch and anterior basal lift. The immediate effect of both techniques was recorded. At first, the patient was taken in the supine position. The upper border of the ribs was palpated, uncovering the patient's chest, and the pressure was applied in a downward direction using two fingers. The stretch was maintained for 10 seconds. The pressure was applied to bilateral ribs. Ten repetitions were given in three sets with a rest period of 1 minute. Followed by this technique, an anterior basal lift was performed. Therapist’s both the hands were placed beneath the posterior ribs and lifted upwards gently. Similar to the first technique, 10 repetitions were given of three sets with a rest period of 1 minute was given. Taking into consideration the patient's general health status, PNF techniques were associated with respiratory and relaxation training.

## Results

The Student's paired t-test was used for the statistical analysis, and SPSS volume 24.0 (IBM Corp., Armonk, NY) was used for the study. The significance level was set at P- 0.05.

A total of 59 patients with high flow oxygen support were willing to participate in the study. There were a total of eight patients from the age group 21-30 years (13.56%), 20 patients from 31-40 years (33.90%), 15 patients from 41-50 years (25.42%), 10 patients from 51-60 years (16.95%), and six patients from the age group of >60 years (10.17%).

According to the HRCT score, there were 37 patients, affected mildly (0-8), on the CT severity score. Twenty-two patients were affected moderately (9-15), whereas no patient was affected severely.

The mean value of heart rate immediately after the intervention was shown as 99.59 and pre-intervention was 90.13 which has a mean difference of 9.45±5.27 which is suggestive of a statistically significant increase in heart rate immediately after intervention taken into consideration the P=0.0001, which was calculated using Student’s paired t-test in all age groups (Table 1).

**Table 1:** Comparison of heart rate at pre- and post-treatment

	Mean	N	Standard deviation	Standard error	Mean difference	t-value
Pre-treatment	90.13	59	9.98	1.30	9.45±5.27	13.77, P=0.0001, S
Post-treatment	99.59	59	9.35	1.21		

The mean value of respiratory rate immediately after the intervention was shown as 22.91 and pre-intervention was 19.18 with a mean difference of 2.93±3.95, suggestive of statistical significant increase in respiratory rate immediately after intervention with P=0.0001, which was calculated using Student’s paired t-test (Table 2).

**Table 2:** Comparison of respiratory rate at pre- and post-treatment

	Mean	N	Standard deviation	Standard error	Mean difference	t-value
Pre-treatment	19.98	59	3.23	0.42	2.93±3.95	5.69, P=0.0001, S
Post-treatment	22.91	59	2.21	0.28		

The mean value of SPO2 immediately after the intervention was shown as 92.76 and pre-intervention was 94.64 which has mean difference of 1.88±1.67 which is suggestive of statistical significant improvement in SPO2 immediately after intervention with P=0.0001, which was calculated using Student’s paired t-test (Table 3).

**Table 3:** Comparison of SPO2 at pre- and post-treatment

	Mean	N	Standard deviation	Standard error	Mean difference	t-value
Pre-treatment	92.76	59	1.74	0.22	1.88±1.67	5.69, P=0.0001, S
Post-treatment	94.64	59	1.72	0.22		

## Discussion

In this study, patients who were tested positive for COVID-19 and admitted in the Inpatient Department were given the treatment with the chest PNF technique to see how it affected the vital signs and level of oxygen saturation. Dangi et al. [10] have studied the effectiveness of chest PNF technique in comparison to breathing exercise in stable COPD patients, and concluded that both the techniques are equally effective in improving the grade of dyspnea and functional capacity. Also, these showed an improvement in pulmonary function and exercise tolerance, supporting the results of this present study where patients have shown improvement in the level of oxygen saturation, which in turn leads to better compliance to further exercises, leading to improved respiratory function. Chest PNF has been proven to be beneficial in a variety of patients who underwent thoracic and abdominal surgeries, pre-operatively, and managing post-operative complications. Various chest PNF techniques encourage continuous inspiration, and enhance ventilation/perfusion mismatch and alveolar-PaO2 gradient. This reduces intrapulmonary shunting and atelectasis risk [11-15]. However, recent studies and available literature are still in dilemma regarding the use of chest PNF techniques in COVID-19 patients. Lack of evidence makes it arduous to comment on the use of chest PNF techniques such as anterior basal stretch lift and intercostal stretch in this population. This study, on the other hand, shows that the chest PNF technique should be used as a part of inpatient rehabilitation for COVID-19 patients with mild to moderate cases.

## Conclusions

This research study draws the conclusion that chest PNF procedures like intercostal stretch and anterior basal stretch lift have an immediate positive influence on oxygen saturation in COVID-19 patients. The chest PNF technique has proven to be quite effective for patients receiving inpatient COVID rehabilitation. In order to achieve the maximum possible physical health, this study explains the treatment strategy and value of exercise compliance in COVID-19 patients.
